# Non-*Helicobacter Pylori Helicobacter* Species in Humans: What Do We Know and Where Should We Go?

**DOI:** 10.34172/mejdd.3375

**Published:** 2026-04-30

**Authors:** Romina Rezaei, Alireza Sadeghi, Maryam Hasanzarrini, Hossain Salehi, Amir Mohammad Salehi

**Affiliations:** ^1^Student Research Committee, Hamadan University of Medical Sciences, Hamadan, Iran; ^2^Clinical Research Development Unit of Shahid Beheshti Hospital, Hamadan University of Medical Science, Hamadan, Iran; ^3^Farhikhtegan Medical Convergence Sciences Research Center, Farhikhtegan Hospital, Tehran Medical Sciences, Islamic Azad University, Tehran, Iran; ^4^Fetal & Pediatric Cardiovascular Research Center, Children’s Medical Center, Tehran University of Medical Sciences, Tehran, Iran

## Dear Editor,

 Shortly after the discovery of *Helicobacter pylori* (*H. Pylori*) by Marshall and Warren in 1982, other *Helicobacter* species have been isolated from the human stomach. These gram-negative spiral-shaped non-*Helicobacter pylori Helicobacter* (NHPH) species mostly colonize animals, including pigs, dogs, and cats. However, five NHPH species have also been identified in the human stomach, including *H. suis, H. felis, H. bizzozeronii, H. salomonis*, and *H. heilmannii s.s*. Among these, *H. suis,* which naturally colonizes pigs and non-human primates, is the most frequently detected NHPH in humans. NHPHs transmission from an infected animal host to a human occurs through direct or indirect contact with the animal reservoir, such as dogs, pigs, and cats. Additionally, *H. suis* could be transmitted via the consumption of undercooked or raw pork meat.^1,2^

 While *H. pylori* is a well-established gastric pathogen, affecting more than half of the world’s population and responsible for a wide spectrum of gastric disorders, NHPHs are detected in a significant proportion of patients with chronic gastritis, peptic ulcers, and mucosa-associated lymphoid tissue (MALT) lymphoma, especially in cases of negative *H. pylori* infection. Studies report varying prevalence rates of gastric NHPHs among patients. While studies report a 0.2%-6% prevalence of gastric NHPHs in unselected symptomatic populations, more recent research from Western countries and Japan indicates a significantly higher prevalence—exceeding 20%—specifically among patients who are *H. pylori*-negative.^1,3,4^

 Due to the overlapping clinical symptoms (epigastric pain, dyspepsia, nausea, vomiting) and histological findings, clinical differentiation between NHPH and *H. pylori *is challenging.^5^ Endoscopic findings of NHPH species are not as widely known as those of *H. pylori*. However, advances in image-enhanced endoscopy (IEE), such as linked-color imaging (LCI) and blue-laser imaging (BLI), have improved the visibility and detection of characteristic features. Several endoscopic features, such as white marbled appearance, cracked-like mucosa, spotty redness, and nodular gastritis-like appearance, have been frequently reported in cases of NHPH gastritis.^6^

 Traditional histological methods for detecting *H. pylori* have limited diagnostic accuracy in detecting and differentiating NHPHs due to morphological resemblance and low bacterial load. Therefore, targeted molecular diagnostic methods, such as polymerase chain reaction (PCR), are used to detect *Helicobacter* spp. DNA and differentiate among species with high sensitivity and specificity.^5^ Other diagnostic methods currently used to identify NHPHs include histopathological and immunohistochemical findings, culture tests, and enzyme-linked immunosorbent assays.^7^

 At present, there is no standardized guideline for eradicating NHPH, and treatment approaches are often empirically based on regimens used to eradicate *H. pylori*, which include broad-spectrum antibiotics and proton pump inhibitors (PPIs). A study showed that a triple-drug combination therapy containing amoxicillin, clarithromycin, and a PPI for 7 days could be a suitable first-line treatment for NHPH as well as *H. pylori* infection.^8^

 NHPH species represent an under-recognized group of gastric pathogens with significant clinical implications, yet current studies on NHPH face several limitations. Most of the studies conducted are single-center and have small sample sizes. Also, a significant number of studies had a retrospective design, which are more prone to biases.^8^ Many aspects of epidemiology, pathogenesis, diagnosis, and proper treatments remain unclear. Therefore, we suggest further comprehensive research to better define the role of NHPH in gastric diseases, improve diagnostic accuracy, and develop an optimal treatment strategy.
